# Recent Advancement in Mangrove Forests Mapping and Monitoring of the World Using Earth Observation Satellite Data

**DOI:** 10.3390/rs13040563

**Published:** 2021-02-05

**Authors:** Chandra Giri

**Affiliations:** Office of Research and Development, United States Environmental Protection Agency, 109 T.W. Alexander Drive, Research Triangle Park, NC 27709, USA

Mangrove forests are distributed in the inter-tidal region between the sea and the land in the tropical and subtropical regions of the world largely between 30° N and 30° S latitude. The total mangrove forest area of the world in the year 2000 was 137,760 km^2^ in 118 countries and territories, accounting for less than 1% of total tropical forests of the world (Figure 1) [1]. Prior to this study, accurate, up-to-date, and reliable information on mangrove distribution was not available. The estimates of world mangroves varied from ~110,000 to 240,000 km^2^ [1].

Mangrove forests provide important ecosystem goods and services for human well-being. They are one of the most productive and biologically complex ecosystems in the world. Recent findings suggest that mangroves annually sequester two to four times more carbon compared to mature tropical forests, and store three to four times more carbon per equivalent area than tropic forests.

The protective role of mangrove forests from natural disasters is well recognized. Mangrove forests received special attention after the Asian Tsunami of 2004 and recent natural disasters such as hurricanes and cyclones.

Mangroves are in a constant flux due to both natural and anthropogenic forces. The forests have been declining at a faster rate than inland tropical forests and coral reefs. Anthropogenic causes are responsible for mangrove destruction at present, but relative sea-level rise could be the greatest threat to mangroves in the future. The continued decline of the forests is caused by conversion to agriculture, aquaculture, tourism, urban development, and overexploitation. Predictions suggest that 30–40% of coastal wetlands and 100% of mangrove forest functionality could be lost in the next 100 years if the present rate of loss continues. Therefore, important ecosystem goods and services (e.g., natural barrier, carbon sequestration, biodiversity) provided by mangrove forests will be diminished or lost.

Despite the importance of mangrove forests, reliable, accurate, and timely information on mangrove forests of the world is not available. Mangroves possess a very distinct spectral signature in remotely sensed data, particularly in the spectral range corresponding visible red, near infrared, and mid infrared, thus making it easier to classify compared to other land cover types. Advancement in remote sensing with the availability of higher spatial, spectral, and temporal resolution and availability of historical remote sensing data provides an opportunity to better characterize, map, and monitor mangrove forests.

Recent advancement in remote sensing data availability, image-processing methodologies, computing and information technology, and human resources development have provided an opportunity to observe and monitor mangroves from local to global scales on a consistent and regular basis. Spectral and spatial resolution of remote sensing data and their availability has improved, making it possible to observe and monitor mangroves with unprecedented spatial and thematic detail. Novel remote sensing platforms, such as unmanned aerial vehicles, and emerging sensors, such as Fourier transform infrared spectroscopy and LiDAR, can now be used for mangrove monitoring. Furthermore, it is now possible to store and analyze large volumes of data using cloud computing.

High quality contributions emphasizing (but not limited to) the topic areas listed below were solicited for the special issue:

- Application of aerial ground remote sensing, photography, multi-spectral, multi-temporal and multi-resolution, satellite data, synthetic aperture radar (SAR) data, hyperspectral data, and LiDAR data.
- Application of advanced image pre-processing for geometric, radiometric, and atmospheric correction, cloud removal, image mosaicking
- Application of advanced image classification and validation techniques including supervised and unsupervised classification
- Application of remote sensing to derive spatio-temporal information on mangrove forests distribution, species discrimination, forest density, forest health, mangrove expansion and contraction, and other ongoing changes in mangrove ecosystems

In the last decade or so, significant improvement has been achieved in terms of remote sensing data availability, classification methodologies, computing infrastructure, and availability of expertise. We now have a large amount of data in need of the integration to answer critical science questions. To accomplish this requires the implementation of automated image pre-processing and classification approaches (Figure 2). At present, not everything can be automated, but many steps including pre-processing that normally constitute 50–60% of project time can be automated.

Pre-processing of satellite data should be centralized, whereas classification and image interpretation can be decentralized (Figure 3). However, there should be an inflow of information from centralized to local levels and vice versa.

The recent trend has been to perform image processing using cloud computing such as Google Earth Engine (GEE) and Amazon Web Services (AWS). Using parallel computing, users will have unlimited computer processing capabilities. Moreover, code and classification algorithms can be shared and discussed in the shared platform. The few disadvantages include a lack of full control of the cloud-computing platform, cost, and the fact that documents are not available or fully explained in some cases.

A brief summary of the twelve papers published in this special issue are presented below.

Younes [2] explored and developed a novel, data-driven approach to extract plant phenology of six different mangrove forests across Australia. They used Landsat imagery and Generalized Additive Models (GAMs) to derive phenology. They found that the Enhanced Vegetation Index (EVI) is directly related to leaf production rate, leaf gain, and net leaf production. Leaf production rate was verified using in-situ data, but the leaf gain and net leaf production was verified using published literature data. The authors also found that the EVI has a two-to-three-month lag time to respond to leaf gain in most cases. The paper concluded that satellite imagery can be useful to better understand mangrove phenology.

In a paper by Yancho et al. [3], a new tool was developed called the Google Earth Engine Mangrove Mapping Methodology (GEEMMM) to map and monitor mangrove forests of the world. The GEEMMM is an “intuitive, accessible and replicable approach” developed primarily for non-remote sensing users including coastal managers and decision makers. The tool was developed in a study conducted in the mangrove forests of Myanmar and is based on cloud computing capabilities GEE. Both qualitative and quantitative accuracy assessment were performed to test the tool. The accuracy assessment shows that the tool is suitable for mangrove mapping and monitoring worldwide. The tool may not be that effective to map large mangrove areas. In addition, internet connectivity may present a challenge in running GEEMMM.

Darmawan et al. [4] monitored the mangrove forests before and after the 1997 forest fire, identified the impact of forest and predicted for the future. The authors used Landsat satellite data acquired in 1989, 1998, 2002, and 2015 and integrated the Markov Chain and Cellular Automata model to compute mangrove forest cover change from 1989 to 2015 in Ambilang National Park Banyuasin Regency, South Sumatra, Indonesia. The change data was used to predict mangrove distribution in 2028. The study showed that approximately 9.6% of mangrove forest in the study area decreased from 1989 to 1998 primarily due to the 1997 forest fire. Mangrove forest has increased by 8.4% from 1998 to 2002, and 2.3% from 2002 to 2015. Future predictions showed continued increase of mangrove forests from 2015 to 2028 ranging from 27.4% to 31%.

Nabab et al. [5], studied the fifth largest mangrove forest of the world in Niger Delta, Nigeria. The forest is under immense pressure from overexploitation and degradation due to the oil and gas industries. The authors mapped the eight main land cover types using Landsat satellite data and L-band radar data of three epochs. They also examined the forest fragmentation of both healthy and degraded mangrove forests. The study concluded that mangrove forests decreased by 500 km^2^ while built-up increased by 1740 km^2^ from 1988 to 2013. The authors also concluded that the mangrove forests in the study was found to be more fragmented in 2013 compared to 1988. The major challenge in this area however, was the availability of cloud free images.

Toosi et al. [6] examined the applicability of multi-sensor remote sensing data to classify land cover classes in a mangrove ecosystem in Iran. They combined Sentinel-2 and WorldView-2 satellite data and classified eight land cover classes using an upscaling approach. The upscaling approach consists of “(i) extraction of reflectance values from Worldview-2 images, (ii) segmentation based on spectral and spatial features, and (iii) wall-to-wall prediction of the land cover based on Sentinel-2 images.” They concluded that the information generated could be useful for the conservation and sustainable management of mangrove forests in Iran.

Quang et al. [7] examined the performance of four different image classification algorithms: Artificial Neural Network (ANN), Decision Tree (DT), Random Forest (RF), and Support Vector Machine (SVM). All four classification approaches are machine learning supervised classification approaches. They used Landsat, SPOT-7 and Sentinel-1 satellite data to classify mangrove forests of Red River estuary of northern, Vietnam. The authors mapped mangrove forest cover change, and age and species composition. The change analysis showed that the mangrove forest area increased from 1975 to 2019 due to successful plantation and forest protection efforts led by local community. The study concluded that SVM was the most accurate classifier out of four classifiers tested. This study concluded that SVM classifier will be valuable for monitoring mangrove plantation projects.

Biswas [8] developed a new method to delineate individual mangrove patches using Aerial Photography with a spatial resolution of 0.08 m, acquired in January 2017. The study was conducted in an area located adjacent the “Everglades National Park, in Florida, USA. This new method utilizes marker-based watershed segmentation. This segmentation methods detects markers using a “vegetation index and Otssu’s automatic thresholding”. The authors used fourteen vegetation indices. The Vegetation Index Excess Green (ExG) without shadow removal produced the most accurate results to detect individual mangrove patches and to detect individual trees.

Zhu et al. [9] estimated the Aboveground Biomass (AGB) of mangrove plantation forests in China. The authors used optical and radar a data obtained from Chinese satellite and Unmanned Aerial Vehicle (UAV) data. The optical data obtained from Geofen-2 (GF-2), SAR data obtained from Geofen-3 (GF-3), and UAV-based Digital Surface Model (DSM) data were used to estimate AGB of Qi’ao Island, China. Random forest classifier and collected field plot data were used for the classification and results validation. The study showed highest accuracy of AGB estimation when all three optical, SAR, and DSM were used. The lowest accuracy was achieved when only optical data was used, higher accuracy was achieved when both optical and SAR data were used. The paper highlighted the importance of combining multi-source data to improve the classification accuracy.

Hu et al. [10] used a combination of ground inventory data, spaceborne LiDAR, optical imagery, climate surfaces, and topographic maps to produce a global AGB map of the world for the year 2004 at 250-m resolution. Image classification was performed using random forest classification method. Training and validation data were obtained from published literature and free-access datasets. The study concluded that the average global mangrove “AGB density was 115.23 Mg/ha, with a standard deviation of 48.89 Mg/ha”. Total AGB storage of global mangrove forest was 1.52 Pg. This result was comparable to other AGB data of the mangrove forests of the world estimated using remotely sensed data. The new biomass map prepared during this study could help understand the global distribution of AGB at 250 m spatial resolution.

Pham et al. [11] investigated the usefulness of gradient boosting decision tree classification approach to estimate Above-Ground Biomass (AGB) of mangrove forests. This study was conducted in Can Gio Biosphere research in Vietnam. A synergistic use of optical and SAR data and a new gradient boosting regression technique called the extreme gradient boosting regression (XGBR) algorithm. The model results were verified using 121 sampling plot data collected during the dry season. Data fusion techniques were used to handle Sentinel-2 multispectral instrument (MSI) and the dual polarimetric (HH, HV) data of ALOS-2 PALSAR-2. Among all models, the XGBR model was the most accurate. The study demonstrated that the XGBR model and remotely sensed data such as Sentinel-1 and ALOS-2 PALSAR-2 data can accurately estimate the AGB of the study area.

Chamberlain et al. [12] combined remote sensing change analysis approach and conventional method of change detection to detect subtle transformations of land cover modification in a large estuarine region of Queensland, Australia. Landsat satellite data acquired in 2004, 2006, 2009, 2013, 2015 and 2017 were used for the classification and change analysis. Image classification was performed using supervised classification method and Maximum Likelihood clustering algorithm. Post classification change analysis was performed. Results from this study showed a steady decline (1146 ha), of mangrove from 2004 to 2017. They found a decreasing trend in the “vegetation extent of open forest, fringing mangroves, estuarine wetlands, saltmarsh grass, and grazing areas, but this was inconsistent across the study site”. Results obtained from this study is expected to be useful to better understand the coastal ecosystem dynamics.

Hauser [13] used cloud computing capabilities of GEE and entire Landsat -7 and Landsat-8 archives to compute spatio-temporal dynamics of mangrove forests and land use changes. This study was conducted in Ngoc Hien District, Ca Mau province in the Mekong Delta of Vietnam. The Classification and Regression Trees (CART) classification method was used to classify (1) dense mangrove forest, (2) sparse mangroves, (3) aquaculture/waterbodies, and (4) built-up and barren lands, land cover classes. The study revealed that the annual rate of deforestation in the study area from 2001 to 2019 was 0.01%. This study contributes to the growing body of literature dealing with dense time series satellite data and cloud computing.

The twelve papers published in this special issue use a wide variety of satellite data and classification approaches to answer important mangrove conservation and management questions. The primary objective is to improve our scientific understanding on the distribution and dynamics of mangrove forests in different parts of the world. These studies help advance our scientific understanding of how various types of remotely sensed data can be utilized with different types of classification approaches to derive meaningful mangrove data and information in support of furthering the science needed to support a global monitoring effort.

## Figures and Tables

**Figure 1.**
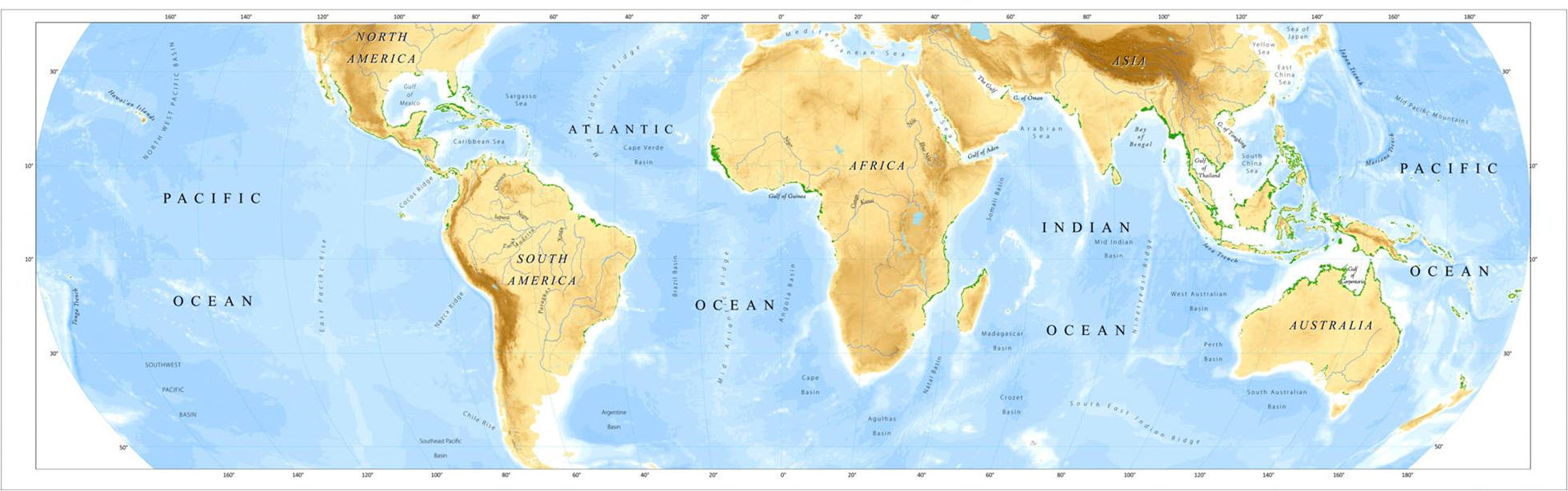
Distribution of the mangrove forests (green) of the world for the year 2000 at 30 m spatial resolution [1].

**Figure 2.**
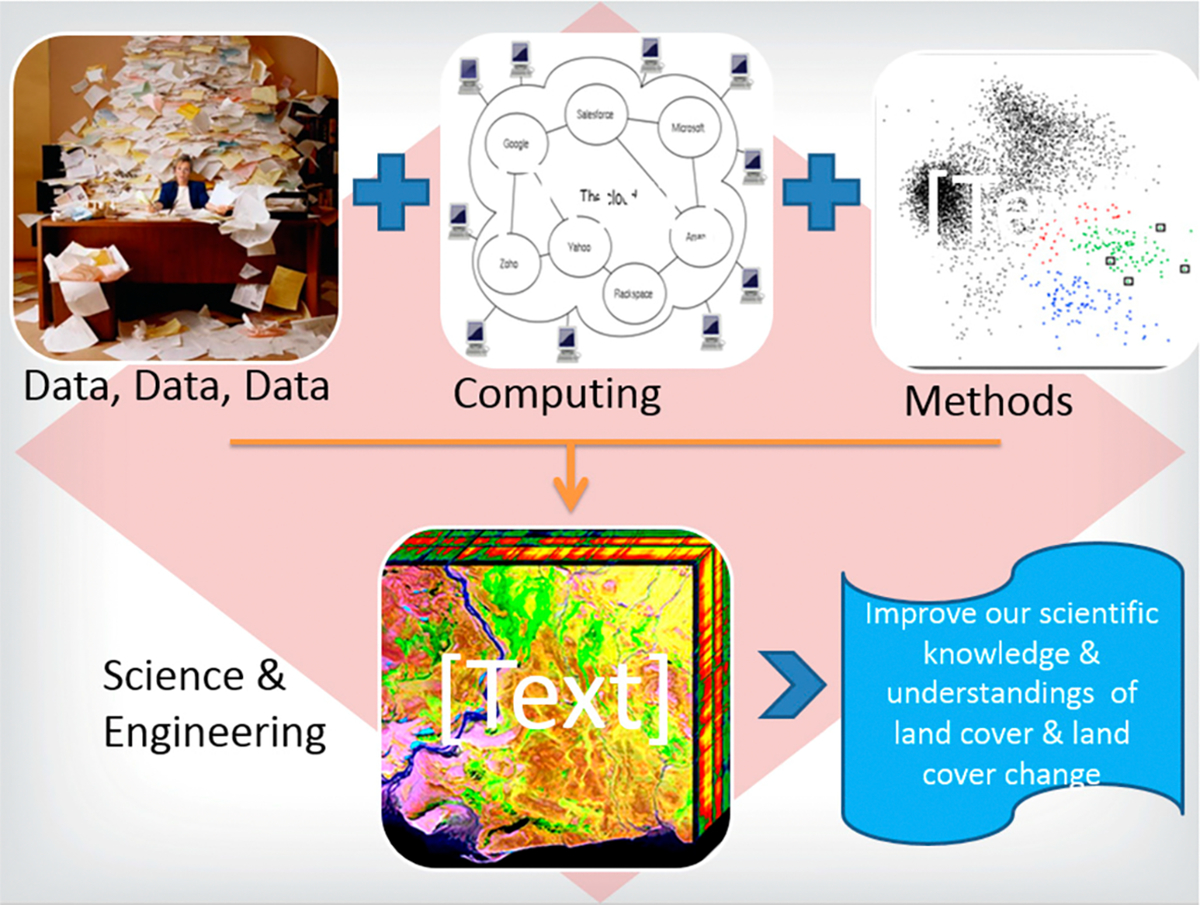
Conceptual diagram of the integration of data, computing, and methods using science and engineering to improve our scientific understanding of mangrove forest cover change.

**Figure 3.**
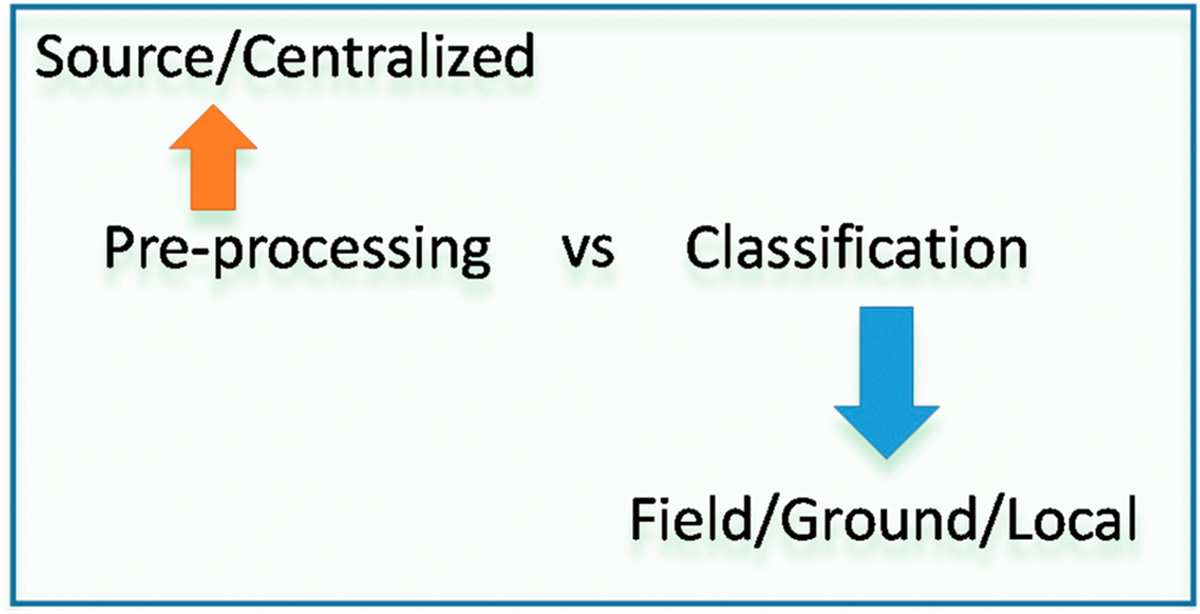
Conceptual framework of pre-processing and image classification showing centralized versus field/ground/local level processing.
